# Preferred question types for computer-based assessment of clinical reasoning: a literature study

**DOI:** 10.1007/s40037-012-0024-1

**Published:** 2012-10-02

**Authors:** Lisette van Bruggen, Margreet Manrique-van Woudenbergh, Emely Spierenburg, Jacqueline Vos

**Affiliations:** 1 Center for Research and Development of Education, University Medical Center Utrecht, P.O. Box 85500, 3508 GA Utrecht, the Netherlands; 2 Erasmus Medical Center, Rotterdam, the Netherlands; 3 Academic Medical Center, Amsterdam, the Netherlands

**Keywords:** Computer-Based Assessment, Clinical reasoning, Medical undergraduate students

## Abstract

Clinical reasoning is a core competence of doctors. Therefore, the assessment of clinical reasoning of undergraduate students is an important part of medical education. Three medical universities in the Netherlands wish to develop a shared question database in order to assess clinical reasoning of undergraduate students in Computer-Based Assessments (CBA). To determine suitable question types for this purpose a literature study was carried out. Search of ERIC and PubMed and subsequent cross referencing yielded 30 articles which met the inclusion criteria of a focus on question types suitable to assess clinical reasoning of medical students and providing recommendations for their use. Script Concordance Tests, Extended Matching Questions, Comprehensive Integrative Puzzles, Modified Essay Questions/Short Answer Questions, Long Menu Questions, Multiple Choice Questions, Multiple True/False Questions and Virtual Patients meet the above-mentioned criteria, but for different reasons not all types can be used easily in CBA. A combination of Comprehensive Integrative Puzzles and Extended Matching Questions seems to assess most aspects of clinical reasoning and these question types can be adapted for use in CBA. Regardless of the question type chosen, patient vignettes should be used as a standard stimulus format to assess clinical reasoning. Further research is necessary to ensure that the combination of these question types produces valid assessments and reliable test results.

## Introduction

Clinical reasoning is a core competence of a doctor and therefore a very important part of medical education. The Academic Medical Center Amsterdam (AMC), the Erasmus Medical Center of Rotterdam (Erasmus MC) and the University Medical Center of Utrecht (UMCU) assess clinical reasoning mainly through written exams. Developing good questions is time-consuming for teachers and staff, as is grading of open answer questions, especially when large groups of undergraduate medical students (>400) take part in the examination.

This study on preferred question types is part of a project of which the main objective is to reduce workload for teachers and staff. The three medical universities mentioned collaborate in developing a shared database of questions that measure clinical reasoning, are constructed according to accepted standards and are suitable for use in Computer-Based Assessments (CBA). Creating a shared question database will reduce workload because individual teachers need to develop less questions and the use of CBA will make it possible to automatically rate exams. Besides making automatic grading possible, CBA also offers the opportunity to produce more authentic patient vignettes by adding materials such as photos, videos and sounds. Another advantage is that CBA provides possibilities for formative and more frequent tests.

### Research questions that we will attempt to answer

The main question to be answered in this literature study is:

*What are the preferred question types to assess clinical reasoning of large groups of undergraduate students with computer*-*based assessments*?

To answer this question several sub questions also need to be answered:

1. What is clinical reasoning and what assessable aspects does it contain?
2. What question types are known to assess clinical reasoning and what specific aspects of clinical reasoning do these question types assess?
3. Which of those question types are suitable -or can be adapted- for CBA?
4. What is known about the psychometrical values of these question types?
5. What is known about construction requirements of these question types?
6. What is known about time investment for both teachers (construction and correction) and students (answering)?

### Definition of clinical reasoning

In the literature many different definitions on clinical reasoning can be found [1–3]. In view of the practical scope of this review we chose a definition that is a synthesis of possible descriptions of clinical reasoning: *Clinical reasoning is the process through which the physician identifies a patient’s most likely diagnosis and determines further policy* [2]. In this process earlier experiences (intuition) as well as analytical skills are important [4]. Beullens et al. [5] describe the distinction between ‘forward reasoning’ (generating a hypothesis or diagnosis based on diagnostic findings) and ‘backward reasoning’ (confirming a hypothesis or diagnosis by interpreting findings). ‘Forward reasoning’ is often used by experts, ‘backward reasoning’ by novices. Beullens et al. note that a person can be an expert on one domain, but a novice on another. The situation and pre-knowledge of a topic determine which strategy is used.

(Bio)medical knowledge is needed to successfully conclude a clinical reasoning process [1, 6]. To answer a clinical reasoning question, students should have underlying knowledge and be able to apply this knowledge to solve a patient’s problem [7]. This principle is also known as the use of illness scripts [3].

Assessing clinical reasoning involves evaluating aspects such as pattern recognition (based on illness scripts), relevant knowledge (on circumstances and pathophysiological disturbances that can lead to a particular disease), complaints and symptoms of diseases and the best treatment and the *ability of hypothetico*-*deductive reasoning* [2].

Schuwirth and Van der Vleuten highlight the fact that whatever question type is used the *stimulus format* (i.e. the content of the question) is more important in determining what is tested than the *response format* (the way the question has to be answered). In practice, however, the response format is often leading in test construction, both in open-ended questions and in MCQ [8]. To test (aspects of) clinical reasoning, it is important according to Schuwirth and Van der Vleuten that the stimulus format is a short patient vignette containing all relevant and uninterpreted data, such as symptoms, research and findings. Based on this patient vignette or stem, students answer a few questions on key decisions. The response format may vary depending on the content of the question. To answer the question, data from the case should be essential [8]. Questions constructed according to this principle are named *Key Feature Questions* (KFQ) in which a *key feature* is defined as a critical step in solving the problem [8, 9]. Adding material such as photos, videos and sounds to patient vignettes can make these vignettes more authentic. This is an important advantage of the use of CBA. However, developing patient vignettes is time consuming. Inexperienced teachers might need up to 3 hours to develop one question according to the *key feature* principle. More experienced teachers are able to develop approximately four questions in an hour. Therefore, questions are expensive to develop [10].

### Question types to assess clinical reasoning

From literature and experience on assessment it is known that there are several question types or assessment formats that are used to assess clinical reasoning in medical education. A literature search on the following question types was conducted:

1. Script Concordance Test (SCT)
2. Extended Matching Question (EMQ)
3. Comprehensive Integrative Puzzle (CIP)
4. Modified Essay Question (MEQ) and Short Answer Question (SAQ)
5. Long Menu Question (LMQ)
6. Multiple Choice Question (MCQ)
7. (Multiple) True/False Question (MTFQ)
8. Virtual Patient (VP)

## Method

In order to answer the first subquestion, current literature on clinical reasoning was studied.

To answer the main research question and subquestion a literature search was carried out in ERIC on 19 November 2010. The terms ‘clinical reasoning’ and ‘medical education’ were used. This search resulted in 42 hits of which 17 were considered relevant. After studying the abstracts of these 17 articles three articles met the inclusion criteria. But after reading them, they were not considered to be relevant.

**Fig. 1:**
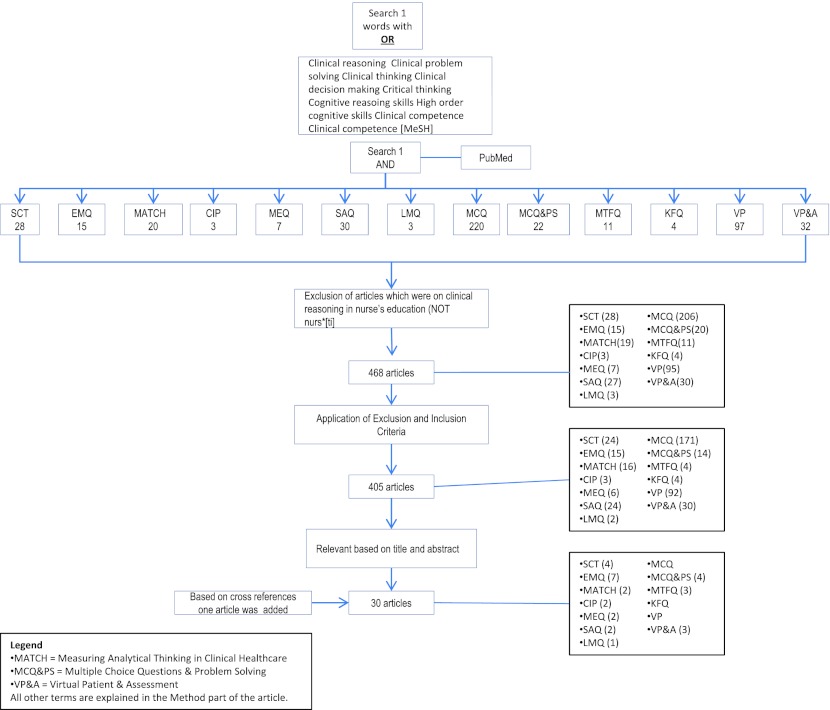
Scheme showing literature search and results in PubMed

A second search was carried out in PubMed on 8 February 2011. The MeSH term ‘clinical competence’ was used. This term was combined with ‘clinical reasoning’ and synonyms such as ‘clinical problem solving’ and ‘clinical thinking’. Next the search string for clinical reasoning was combined with question types found in the literature: Script Concordance Tests, Comprehensive Integrative Puzzles, Extended Matching Questions, Short Answer Questions, Long Menu Questions, Multiple Choice Questions and Virtual Patients. Besides these questions Oral Exams and OSCE are used to assess students on clinical reasoning, often combined with testing communication and physical examination skills. Oral Exams and OSCE fall outside the scope of this literature study since CBA is not possible in this context. The search on ‘Multiple Choice Questions’ and ‘Virtual Patients’ was further limited by adding the term ‘problem solving’ for MCQ and ‘assessment’ for VP. Fig. 1 shows the results of the search in PubMed.

Articles had to meet the following inclusion criteria:

- Focus on question types specific for assessment of clinical reasoning
- Assessment of medical students ((under)graduate and postgraduate)
- Recommending use and providing construction guidelines for questions types

Excluded were articles:

- With a focus on clinical reasoning of nurses; NOT nurs*[ti]
- In languages other than Dutch or English
- Published before 1990
- Not accessible in full text via the AMC, Erasmus MC or UMCU

Articles that met the inclusion criteria were screened for relevance by their titles and abstracts. Remaining articles were studied to answer the research question. References of these articles were also screened for relevance, following the same inclusion and exclusion criteria.

Excluding the articles that were not accessible in full text might be a potential confounder of the results, but the authors are confident that no critical information was missed. One essential article on EMQ that was not accessible in full text via the AMC, Erasmus MC or UMCU was purchased [11].

## Results

The results of the literature study indicate that all question types mentioned are suitable to assess -aspects of- clinical reasoning, but not all question types are suitable for use in CBA. The characteristics of the question types are described in the following paragraph.

### Script Concordance Test (SCT)

SCT [7, 12–16] focuses on data interpretation by asking for probabilities in a specific context, based upon new signs and symptoms. SCT therefore tests knowledge of illness scripts. The possibility of score differentiation gives SCT a high discriminatory quality which increases reliability. A panel of at least 10 experts is needed to decide on the different degrees of probability of a certain diagnosis [13]. SCT is mainly applied in assessment of postgraduate students and residents. Sibert et al. [7]. mention the use of SCT in a web-based test. Students can answer 16 case histories with 145 questions in one hour.

### Extended Matching Question (EMQ)

EMQs [5, 10, 11, 17–20] consist of a theme, a lead-in statement, a list of a minimum of 6 and a maximum of 26 options and two or more cases. The focus is on taking decisions in the clinical process, taking into account diagnostics, diagnosis and therapy. EMQs test application of knowledge rather than isolated facts; Beullens et al. [5] prove that an exam with EMQs tests (an aspect of) clinical reasoning. The response format with a large option list minimizes cuing. Hundred EMQs are needed for a reliable and valid exam. Students can answer 24 EMQs with three stems in one hour. EMQ can be used in CBA.

### Comprehensive Integrative Puzzle (CIP)

A CIP [21, 22] consists of between four up to seven rows and columns. A CIP focuses on combining given options for medical history, physical examination, additional tests and therapy in order to construct coherent case histories for given diagnoses. By completing a CIP students construct illness scripts, which is an important part of clinical reasoning, and thus tests backward reasoning [2]. The CIP has a highly discriminatory quality because of the possibility of score differentiation. Experienced teachers develop a CIP in 1–2 h. Students can finish four to five CIPs in one hour.

### Modified Essay Question (MEQ) and Short Answer Question (SAQ)

Both MEQ and SAQ [8, 10, 23–25] pose questions based on patient vignettes. Students answer open questions on diagnoses and proposals of therapy. MEQ and SAQ have a high discriminatory quality because of the possibility of score differentiation and produce tests with a high reliability. Correcting and scoring of MEQ and SAQ is time consuming. Rademakers et al. [25] mention 4.7 h per question with student numbers not exceeding 400. Automatic rating of open-ended questions is not yet possible.

### Long Menu Question (LMQ)

LMQ [9] focuses on decision taking in the clinical process regarding diagnostics, diagnosis and therapy. Students type a word and a scroll down menu appears from which options can be chosen. This response format minimizes cuing, but no evidence was found in the literature for reliability and discriminatory capacity. Developing questions in a LMQ format is only possible if students answer the question with one single word, due to the fact that sentences cannot be alphabetically arranged in a computer program. A prerequisite for the effective use of LMQs is the availability of long lists of possible alternatives, which makes the construction of LMQ time consuming.

### Multiple Choice Question (MCQ)

If combined with a patient vignette that contains the information needed to answer the question, a MCQ [19, 26, 27] can be used for assessing clinical reasoning. The focus may be on decision making in the clinical process regarding diagnostics, diagnosis and therapy. However, due to time constraints teachers tend to develop MCQs without sufficient context that only test factual knowledge. Students can answer 30 MCQs in an hour. MCQs can easily be used in CBA.

### (Multiple) True/False Question (MTFQ)

In MTFQ [10, 28, 29] students judge the truth of statements or conclusions in the clinical reasoning process. There are serious doubts on the validity of this question type. Student scores are influenced by exam techniques and willingness to take risks. Research indicates a gender bias in favour of men regarding the question mark option [28]. There is a considerable chance of a correct answer based on the wrong information. A minimum of hundred MTFQs generate reliable test results. MTFQ can be used in CBA.

### Virtual Patient (VP)

VP [30–32] focuses on going through a complete process of clinical reasoning until the virtual patient is cured or dies. Content validity and reliability are low due to the low degree of representativeness of the test compared with the subject matter; a considerable amount of time is needed to complete one VP. VPs are very expensive to develop. Special software and hardware is needed to implement VPs in education. No data were found in literature on the time students need to answer a VP.

From this description it follows that the question types differ in focus on which aspect of clinical reasoning they assess. SCT, EMQ, CIP, MCQ, MTFQ can be used -or adapted for use- in CBA. For MEQ and SAQ only limited digital correction is possible; to implement VP as an assessment tool special software and hardware are needed. These three question types are therefore currently not suitable for use in CBA.

## Discussion

This literature study aimed to answer the question as to which question types are preferred for use in a CBA of clinical reasoning for large groups of undergraduate medical students.

SCT and CIP yield higher discriminatory values and therefore better distinguish between prepared and unprepared students. Higher discriminatory values of questions increase the reliability of the test where fewer questions are needed. However, in the literature studied on SCT there is no mention of assessment of undergraduate students using this question type. The need for an expert panel for SCTs is labour intensive and therefore does not reduce the workload of teachers. It is not yet possible to organize an expert panel in CBA. SCTs cannot be recommended for use in CBA.

The validity of MTFQ and VP for assessment of clinical reasoning is doubted or considered low. MTFQ are no longer used by the National Board of Medical Examinations in the USA (NBME) and the Dutch Interuniversity Medical Progress test (iVTG), due to several types of bias.

The current scoring system in CBA necessitates constructing completely right or completely wrong options for EMQ, MCQ and LMQ. In clinical practice there is often no 100 % right or wrong. Even experts may have different opinions on approach or policy. Current paper-based exams do not always reflect this nuance in clinical practice. In closed-question exams, answers are rated either right or wrong. Using a scoring system with weighted answers, as is common with SCT, better reflects clinical practice. In these scoring systems the score of the student reflects the resemblance of his answer to the answers given by a panel of experts. Further research into the use of differentiated scoring rules in CBA is recommended.

Using a mix of question types within a test offers possibilities to broadly test clinical reasoning. A mix of question types also increases the validity of the test regarding the assessment of the clinical reasoning capacity of students. Besides, the use of fixed question formats will help teachers to construct good questions and will therefore increase the construct validity of the questions. Schuwirth and Van der Vleuten [8] underline this by stating that using a combination of existing question types is more effective than searching for or developing a new panacea.

## Conclusion

Based on the findings in literature on validity, reliability, discriminatory power, adaptability for CBA and practical use, the authors consider a combination of CIP and EMQ most suitable for a CBA on clinical reasoning for large groups of undergraduate medical students. A patient vignette containing the information needed to answer the questions posed is an indispensable part of both question types. Therefore, while constructing these question types particular attention should be given to developing good patient vignettes according to the key feature principle. This patient vignette is indeed the stimulus format that makes actual assessment of clinical reasoning possible. Therefore training teachers in developing patient vignettes is essential. Developing a weighted scoring system in CBA is recommended. A digital format for both CIP and EMQ needs to be developed. Further research is necessary to ensure that CIP and EMQ in a CBA produce valid and reliable tests.

## Essentials

- Use a combination of CIP and EMQ in CBA of clinical reasoning.
- Training teachers in writing good patient vignettes is recommended.
- CBA makes it possible to construct more authentic patient vignettes.
